# Dietary intake characteristics in older adults: A systematic review of physical, social, psychological, and behavioral limitations

**DOI:** 10.1016/j.jarlif.2026.100064

**Published:** 2026-02-06

**Authors:** Kaori Kinoshita, Kanae Furuya, Ryoko Katagiri

**Affiliations:** a Department of Frailty Research, Center for Gerontology and Social Science, Research Institute, National Center for Geriatrics and Gerontology, 7-430, Morioka-cho, Obu, Aichi 474-8511, Japan; b Department of Clinical Nutrition, Faculty of Health Science, Suzuka University of Medical Science, 1001-1, Kishioka-cho, Suzuka, Mie 510-0293, Japan; c Faculty of Informatics, Graduate School of Informatics, Chiba University, 1-33 Yayoi-cho, Inage-ku, Chiba-city, Chiba 263-8522, Japan

**Keywords:** Older adults, Epidemiological studies, Community dwellers, Dietary intake, Nutrients, Food

## Abstract

Older individuals generally have decreased food intake owing to physical, social, psychological, and behavioral limitations. However, the characteristics of food and nutrient intake resulting from these limitations are poorly organized. Organizing knowledge for practical application in older adults is necessary when considering nutrition care strategies. This review aimed to characterize food and nutrient intake according to these exposure factors in older individuals. This systematic review followed the Preferred Reporting Items for Systematic Review and Meta-Analysis guidelines (PROSPERO, CRD42024582151). MEDLINE and Web of Science were searched in September 2024 using the following inclusion criteria: community-dwelling older adults aged ≥65 years (participants); factors contributing to physical, social, psychological, or behavioral limitations affecting food and nutrient intake (exposure); food and/or nutrient intake, dietary patterns, and overall diet quality (outcomes); and cross-sectional and cohort studies (study design). Among the 2,354 studies screened, 29 studies were analyzed. These included studies addressed the following exposure factors: oral function and physical activity (physical); economic status, marital status, household size, and educational attainment (social); depressive symptoms (psychological); and food security, nutritional knowledge, and cooking skills (behavioral). Reduced oral function was associated with lower intakes of meat, fish, legumes, vegetables, and fruits. Low economic status and educational level were associated with poor diet quality. However, there was limited research characterizing the dietary intakes of individuals with other factors, such as poor cooking skills or decreased sense of taste and smell. Further research is needed.

## Highlights

• This study reviewed the factors limiting dietary intake in older adults.
• The study aimed to gain insights into unintentionally unhealthy eating habits.
• Poor oral function reduced meat, fish, legume, vegetable, and fruit intakes.
• Few studies clarified the association of other factors with nutrients or foods.
• Further research on the link of multifactorial limitations with diet is needed.

## 1 Introduction

The population of older adults needing support with activities of daily living is projected to increase fourfold by 2050 [1]. Accordingly, measures to address the increasing rate of disability in this population are urgently needed. Meeting nutritional needs is important because malnutrition can lead to hospitalization, disability, and death. However, older adults generally experience decreased food intake and appetite with age [2]. This decline is driven by multifactorial physical (e.g., decreased oral function, physical inactivity, and diminished taste and smell—key factors in sarcopenia and anorexia of aging, positioned within the multidimensional construct of frailty), social (e.g., living alone, social isolation, decreased social participation, and lack of support systems), psychological (e.g., depression and cognitive decline), and behavioral (e.g., decreased shopping ability, limited access to food, and poor cooking skills) limitations [2,3]. These factors often result in unintentional unhealthy eating habits. Therefore, nutritional care for older adults must differ from interventions targeting unhealthy eating driven by personal behaviors (e.g., excessive snacking and picky eating).

Older adults have lower energy requirements than middle-aged adults because of age-related declines in activity, but their micronutrient needs do not decrease proportionally [4]. Thus, micronutrient deficiencies may occur even when energy needs are met. Many micronutrients have antioxidant, anti-inflammatory, and metabolic coenzyme functions and help prevent accelerated aging [5,6]. Therefore, to maintain and improve the health of older adults, it is essential to address not only insufficient energy intake, but also deficiencies in protein and micronutrients. To do so, it is useful to systematically identify nutrients or foods likely to be deficient. However, food and nutrient intake patterns resulting from the physical, social, psychological, and behavioral limitations of older adults are not well organized, and nutritional care should be tailored to these limitations. Organizing such knowledge for practical use in local older adults is necessary when planning dietary management strategies.

Therefore, this systematic review aimed to examine how multidimensional exposure factors related to food and nutrient intake, clarify how physical, social, psychological, and behavioral constraints are associated with intake in older adults, and identify specific dietary support needs. This study systematically organized food and nutrient intake patterns across these four domains among community-dwelling older adults, an analysis not previously conducted, and identified vulnerabilities in specific nutrients and food groups within each domain where evidence was available.

## 2 Methods

### 2.1 Study design and search strategy

This systematic review followed the Preferred Reporting Items for Systematic Reviews and Meta-Analyses (PRISMA) guidelines [7], and the protocol was registered in PROSPERO (CRD42024582151).

MEDLINE and Web of Science were independently searched in September 2024. To obtain the most up-to-date information, the search period was set at five years (1 June 2019 to 1 June 2024). The primary search terms are shown in the **Supplementary material**. After removing duplicate records, two reviewers (K.K. and K.F.) independently screened titles and abstracts against predefined inclusion criteria, followed by an independent full-text review of potentially eligible studies. Disagreements were resolved through discussion, with a senior researcher (R.K.) consulted when consensus was not reached. Factors examined in the included studies were categorized into four domains: physical, social, psychological, and behavioral.

### 2.2 Study selection

Eligible studies focused on community-dwelling adults aged ≥65 years. For studies including participants aged <65 years, only those reporting stratified results for individuals aged ≥65 years were included. Studies targeting specific diseases or institutionalized or hospitalized populations were excluded. Included studies were peer-reviewed, available in full text, written in English, and conducted in human subjects. Eligibility criteria were defined using the PEO(S) framework: P (participants): community-dwelling adults aged ≥65 years; E (exposure): physical, social, psychological, or behavioral factors affecting food and nutrient intake (see **Supplementary material**); O (outcomes): food and nutrient intake, dietary patterns, and overall dietary quality; S (study design): cross-sectional and cohort studies.

### 2.3 Evaluation of risk of bias within studies

Two reviewers (K.K. and K.F.) independently assessed methodological quality using the Newcastle-Ottawa Scale (NOS) [8] for cross-sectional studies. Disagreements were resolved through discussion or consultation with a senior researcher (R.K.).

The NOS is a widely used instrument to evaluate the methodological quality of cohort studies in systematic reviews [8,10]. It assesses studies across three domains: selection of study groups, comparability between groups, and ascertainment of exposure or outcomes. The NOS awards up to nine points, with scores determined by the total number of stars assigned across these domains. In accordance with previous research, study quality was classified as follows: scores of 0–3, low quality; 4–6, moderate quality; and 7–9, high quality [11].

The AXIS tool evaluates multiple aspects of study quality, including design, sample size and characteristics, measurement validity, internal consistency, results, analytical approaches, and limitations [9]. It consists of 20 items grouped into three core domains: reporting quality, methodological rigor, and potential sources of bias. Although AXIS lacks a formal scoring system, thresholds from previous appraisals were applied [[12], [13], [14], [15]]. Each item is scored based on a “yes” response, and total scores are interpreted as follows: >15 points indicates high quality; 10–15 points, moderate quality; and <10 points, low quality [16,17].

## 3 Results

### 3.1 Search results

**Fig. 1:**
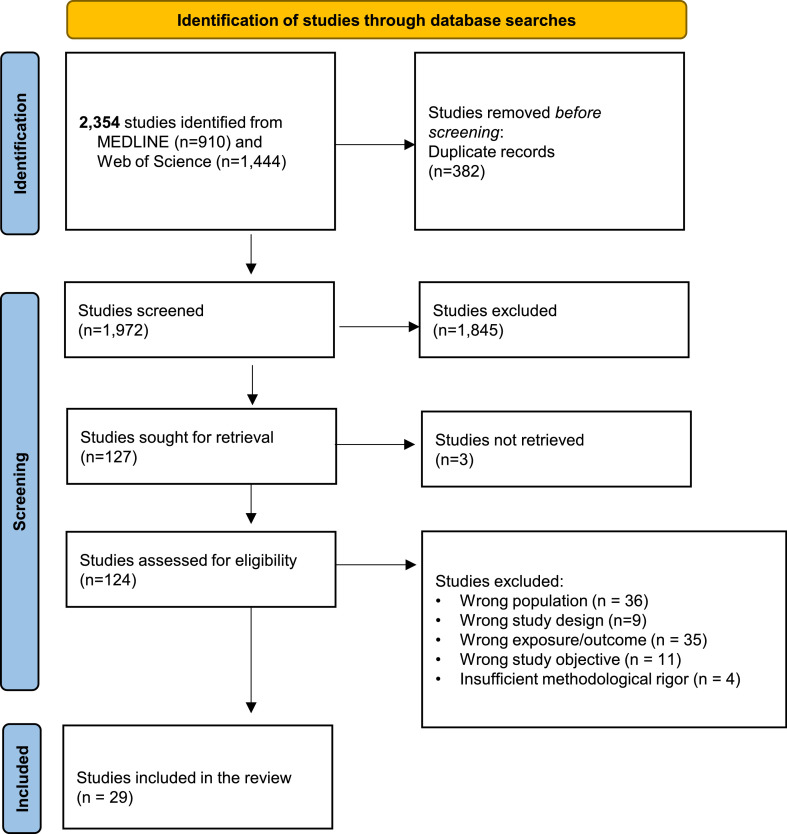
**Study selection flow chart.** PRISMA flow diagram illustrating the study identification, screening, eligibility, and inclusion. A total of 2,354 studies are identified through database searches (MEDLINE, n = 910; Web of Science, n = 1,444). After removing 382 duplicates, 1,972 studies are screened. Of these, 1,845 studies are excluded based on the title and abstract. A full-text assessment is conducted for 127 studies, of which the full-text of 3 studies are not retrieved. Among the 124 assessed studies, 95 studies are excluded due to incorrect population (n = 36), study design (n = 9), exposure/outcome (n = 35), and study objective (n = 11) or insufficient methodological rigor (n = 4). Ultimately, 29 studies are included in the systematic review.

A total of 2,354 articles were identified in the initial database search, of which 382 duplicates were removed. After title and abstract screening of 1,972 articles, 1,845 were excluded for not meeting the selection criteria. Three articles were excluded because full texts were unavailable, leaving 124 articles for full-text review. Of these, 95 were excluded based on the inclusion and exclusion criteria. Fig. 1 shows the study selection flowchart.

The most common reason for exclusion was “wrong population” (n=36), including studies focused on younger individuals or populations with specific diseases, preventing evaluation of outcomes relevant to the target age group. In addition, 35 studies were excluded because of incompatible study designs, either examining different exposure variables or analyzing relationships in the reverse direction (e.g., dietary intake as the exposure and social factors as outcomes). Studies classified as having “insufficient methodological rigor” lacked statistical testing, showed inconsistencies between stated objectives and analytical methods, or demonstrated potential reporting bias.

### 3.2 Study characteristics

A total of 29 studies were included: 25 cross-sectional studies [[18], [19], [20], [21], [22], [23], [24], [25], [26], [27], [28], [29], [30], [31], [32], [33], [34], [35], [36], [37], [38], [39], [40], [41], [42]] and 4 cohort studies [[43], [44], [45], [46]]. Sample sizes ranged from 84 to 85,456 participants (including individuals aged <65 years). Although the literature search included terms related to declines in taste and smell as physical function factors, no studies examined taste or smell as explanatory variables in relation to food or nutrient intake. Many studies on cognitive function treated food and nutrient intake as explanatory variables and cognitive function as the outcome, which did not align with this review’s objectives and were therefore excluded. For social factors, many studies were identified, but most assessed dietary quality rather than specific food groups or nutrient intake. Regarding behavioral factors, only one study examined the association between nutritional knowledge, cooking skills, and food or nutrient intake.

Table 1 presents an overview of the included studies. The studies used various methods to assess dietary quality. Some studies investigated food groups comprehensively, whereas others focused on specific groups (e.g., vegetables and fruits). Some studies focused only on macronutrients, whereas others assessed macronutrients and micronutrients or specific nutrients, such as dietary fiber. Some studies investigated the effects of exposure factors on nutrient deficiency and excess intake.

**Table 1:** Overview of the included studies.

Author, year of publication	Exposure	Outcome (foods/nutrients/quality)	Study design	Country	Sample size, (males %)	Participant age (years)	Results	Covariates
**Physical factors**								
Iwasaki et al., 2024 [18]	Number of functional teeth	DII	Cross-sectional	Japan	2407 (43.7)	45–98	In participants aged ≥75 years, a higher number of functional teeth is associated with a lower DII (i.e., diet with greater anti-inflammatory potential; β=-0.94, 95% CI=-0.171, -0.018).	Age, sex, occupation type, smoking status, alcohol consumption, exercise status, BMI, hypertension, diabetes, and number of natural teeth.
Iwasaki et al., 2024 [18]	FTUs	DII	Cross-sectional	Japan	2407 (43.7)	45–98	In participants aged ≥75 years, a higher number of FTUs is associated with a lower DII (i.e., diet with greater anti-inflammatory potential; β=-0.094, 95% CI=-0.17, -0.017).	Age, sex, occupation type, smoking status, alcohol consumption, exercise status, BMI, hypertension, diabetes, and number of natural teeth.
Milledge et al., 2021 [19]	FTUs	Achievement of recommended NRV values for total energy and macronutrients and micronutrients (Na; K; I; P; Ca; Mg; Zn; Fe; dietary folate equivalents; thiamin; niacin; riboflavin; and vitamins A, C, D, and E) is assessed.	Cross-sectional	Australia	608 (0)	78–100	In comparison with the natural FTU only group, the replaced FTU only group is more likely to have poor macronutrient intake (OR: 2.00, 95% CI=1.01, 3.94).	Number of FTUs, age, energy intake, BMI, comorbidity, and post-school education.
Kusama et al., 2023 [20]	Number of teeth with/without dental prostheses	Intake of protein (total, animal, and plant)	Cross-sectional	Japan	2095 (43.9)	≥65	There is no significant difference in total protein intake between participants with ≥20 remaining teeth and those with 10–19 remaining teeth, regardless of whether they have dental prostheses or not (p>0.05). Among those with 0–9 remaining teeth, total protein intake is significantly lower in those without a dental prosthesis (β=-2.31, 95% CI=-3.47, -1.14) than in those with a dental prosthesis (β=-0.47, 95% CI=-0.96, 0.01).	Age, sex, income, education, comorbidities (stroke, diabetes, cancer), housing damage, living arrangement, marital status, ADL, smoking status, and alcohol consumption.
Kida et al., 2023 [21]	Number of teeth	Intakes of protein, fat, saturated fatty acid, carbohydrates, n-3 fatty acids, n-6 fatty acids, total dietary fiber, potassium, calcium, iron, vitamin C, and folic acid	Cross-sectional	Japan	238 (34.5)	≥20	Saturated fatty acid intake is increased in those with fewer teeth (β=-0.148, p=0.043).	Sex, age, and meal sequence (eating meals in the order of “vegetable dishes,” “meat and fish dishes,” and “staple foods”).
Shen et al., 2023 [22]	Number of teeth	DDS, intake frequency of foods (fresh fruits, fresh vegetables, meat, fish and aquatic products, eggs, legumes, preserved vegetables, sugar and sweets, tea, and garlic)	Cross-sectional	China	54796 (41.2)	87.9±11.5	Participants with <20 natural teeth have lower DDSs than those with ≥20 natural teeth (edentulous: β=-0.39, 95% CI=-0.48, -0.30; 1–9 teeth: β=-0.46, 95% CI=-0.55, -0.37; 10–19 teeth: β=-0.36, 95% CI=-0.46, -0.26). For individual food items, edentulous, 1–9 natural teeth, and 10–19 natural teeth are associated with lower odds of regular intakes of fresh fruits, fresh vegetables, meat, fish and aquatic products, eggs, legumes, preserved vegetables, tea, and garlic but higher odds of regular intakes of sugar and sweets.	Cohort, age, sex, ethnicity, level of education, type of residence, married status, income level, smoking status, alcohol intake, regular exercise, and BMI.
Gaewkhiew et al., 2021 [23]	FD	Twelve-month changes in three dietary patterns—healthy, meat-rich, and carbohydrate-rich—are analyzed based on factor loadings of key food groups	Longitudinal	Thailand	651 (27.3)	≥60	In comparison with those with neither FD nor dentures, participants with FD (containing ≥10 occlusal contacts) have larger increases in healthy (β=0.13; 95% CI: -0.13, 0.39) and carbohydrate-rich diet intake (β=0.12; 95% CI: -0.17, 0.40) and larger reductions in meat-rich diet intake (β=-0.12; 95% CI=-0.45, 0.21). However, the differences are not significant.	Baseline age groups, sex, residence area, education, wealth quartiles, smoking status, physical activity, hypertension, hyperlipidemia, diabetes, medications, total energy intake, and dietary pattern score.
Gaewkhiew et al., 2019 [23]	FD with/without dentures	Intakes of energy; carbohydrate; fat; protein; saturated fat; cholesterol; crude fiber; dietary fiber; calcium; iron; and vitamins A, B1, B2, B3, B6, B12, C, and E	Cross-sectional	Thailand	788 (30.0)	60–96	Participants with functional dentition have significantly greater intakes of dietary fiber (β=2.69, 95% CI=0.02, 5.37) and vitamin B1 (β=0.66, 95% CI=0.03, 1.29) than those with neither functional dentition nor dentures.	Age, sex, residence area, education, wealth tertiles, physical activity, hypertension, hyperlipidemia, diabetes, and energy intake.
Lin et al., 2021 [24]	Number of POSAs	Intakes of fruits (5 groups), seafoods and meats (4 groups), vegetables (5 groups)	Cross-sectional	Taiwan	1100 (28.6)	≥65	The greater the reduction in POSAs, the higher the odds ratio for a reduction in the number of “able to eat” fruits (≤3 groups), seafood and meat (≤2 groups), and vegetables (≤4 groups) consumed.	Sex, age, educational level, number of natural teeth, and removable denture (full or partial).
Lin et al., 2021 [24]	Number of natural teeth	Able to eat fruits (5 foods), seafoods and meats (4 foods), vegetables (5 foods)	Cross-sectional	Taiwan	1100 (28.6)	≥65	In comparison with participants with ≥20 natural teeth, those with <20 natural teeth have a lower number of “able to eat” vegetables (≤4 groups) consumed (OR=2.8, 95% CI=1.44, 5.31).	Sex, age, educational level, POSA (categories by Eichner index), removable denture (full or partial).
Lin et al., 2021 [24]	Removable denture use	Intakes of fruits (5 foods), seafoods and meats (4 foods), vegetables (5 foods)	Cross-sectional	Taiwan	1100 (28.6)	≥65	Removable denture use is not associated with food intake.	Sex, age, educational level, POSA (categories by Eichner index), and number of natural teeth.
Fukutake et al., 2019 [25]	Occlusal force	Intakes of foods (green and yellow vegetables, other vegetables, fruits, fish and shellfish, meats) and nutrients (protein; carbohydrate; and vitamins A, B2, C, α-tocopherol, dietary fiber, folate, n-3 fatty acids, and n-6 fatty acids)	Cross-sectional	Japan	124 (48.2)	69–81	Occlusal force is positively associated with intakes of fish and shellfish (β=0.188, p=0.026).	Age, sex, educational level, self-assessed financial status, and OSA score.
Fukutake et al., 2019 [25]	OSA	Intakes of foods (green and yellow vegetables, other vegetables, fruits, fish and shellfish, meats) and nutrients (protein; carbohydrate; and vitamins A, B2, C, α-tocopherol, dietary fiber, folate, n-3 fatty acids, and n-6 fatty acids)	Cross-sectional	Japan	124 (48.2)	69–81	OSA score is positively associated with intakes of green and yellow vegetables (β=0.160, p=0.031) and α-tocopherol (β=0.161, p=0.031).	Age, sex, educational level, and self-assessed financial status.
Karawekpanyawong et al., 2023 [26]	Masticatory performance	Intakes of energy; protein; carbohydrate; fat; dietary fiber; calcium; and vitamins D, A, C, E, B1, B2, B3, B5, B6, B12, K, γ-tocopherol, zinc, folic acid, iron, β-carotene, n-3 fatty acids, and n-6 fatty acids	Cross-sectional	Japan	84 (46.4)	90	Participants with masticatory performance <173 mg/dL have lower intakes of folic acid and vitamin A than those with masticatory performance ≥173 mg/dL (β=1.500, 95% CI=0.329, 2.670).	Sex, education, higher-level functional capacity, and BMI (≤20 kg/m²).
Karawekpanyawong et al., 2023 [26]	SSFR	Intakes of energy; protein; carbohydrate; fat; dietary fiber; calcium; and vitamins D, A, C, E, B1, B2, B3, B5, B6, B12, K, γ-tocopherol, zinc, folic acid, iron, β-carotene, n-3 fatty acids, and n-6 fatty acids	Cross-sectional	Japan	84 (46.4)	90	SSFR is positively associated with the intakes of vitamins A (β=40.451, 95% CI=6.891, 74.011), B2 (β=0.025, 95% CI=0.004, 0.046), γ-tocopherol (β=0.225, 95% CI=0.048, 0.402), and n-6 fatty acids (β=0.126, 95% CI=0.021, 0.231) and negatively with the intake of carbohydrate (β=-1.140, 95% CI=-1.875, -0.404).	Sex, education, higher-level functional capacity, and BMI (≤20 kg/m²).
Karawekpanyawong et al., 2023 [26]	Number of teeth	Intakes of energy; protein; carbohydrate; fat; dietary fiber; calcium; and vitamins D, A, C, E, B1, B2, B3, B5, B6, B12, K, γ-tocopherol, zinc, folic acid, iron, β-carotene, n-3 fatty acids, n-6 fatty acids	Cross-sectional	Japan	84 (46.4)	90	The number of teeth is positively associated with the intakes of calcium (β=3.284, 95% CI=0.418, 6.150), folic acid (β=2.514, 95% CI=0.909, 4.118), β-carotene (β=39.268, 95% CI=6.304, 72.232), vitamin C (β=0.866, 95% CI=0.132, 1.601), and vitamin K (β=2.027, 95% CI=0.206, 3.848).	Sex, education, higher-level functional capacity, and BMI (≤20 kg/m²).
Kimble et al., 2023 [27]	Dental status and oral health problems (combined)	EDI or HEI	Cross-sectional	UK and USA	UK, 855 (100); USA, 1970 (50)	UK, 78–98; USA, 70–79	Combined measure of dental status and oral health is not associated with diet quality.	UK cohort: age, social class, smoking status, moderate/heavy drinking, low physical activity, and history of cardiovascular disease and diabetes. USA cohort: age, sex, race, level of education, smoking status, low physical activity, and history of cardiovascular disease and diabetes.
Abe et al., 2022 [42]	Social participation (activity)	DVS	Longitudinal	Japan	6168 (49.3)	65–84	Participation in sports groups and hobby groups is associated with a lower RR of unfavorable eating habits (sports groups: RR=0.95, 95% CI=0.90, 1.00; hobby groups: RR=0.93, 95% CI=0.90, 0.97).	Age, sex, living situation (with others or alone), marital status, duration of residence, and social participation (volunteer, sports, hobby groups, senior citizen club, neighborhood association).
**Social factors**								
Vaudin et al., 2021 [28]	Household food expenditure	HEI-2010	Cross-sectional	USA	3056 (49.2)	≥60	Food expenditure (money spent on food per household member in last 30 days on special diet) is associated with diet quality (β=5.4, 95% CI=2.4, 8.5 in males; β=3.4, 95% CI=1.1, 5.7 in females).	Age, race/ethnicity, education, marital status, household size, self-reported health, household food security, and nutrition awareness/use.
Teixeira et al., 2019 [29]	Household income	MDP	Cross-sectional	Portugal	1407 (42.5)	≥65	Household income is not associated with the MDP.	Sex, age, regional area, education, marital status, and residence.
Silva et al., 2019 [30]	Per capita family income in minimum wages	Intake of dietary fibers	Cross-sectional	Brazil	1509 (40.5)	≥60	Higher family income (1–2 and >2 minimum wages) is associated with inadequate dietary fiber consumption (1–2: PR=0.95, p=0.017; >2: PR=0.93, p=0.002).	Sex, age, marital status, number of chronic diseases, physical activity, smoking status, desire to change weight, and sleep hours/day.
Kurotani et al., 2020 [31]	Household income	Japanese Food Guide Spinning Top scores	Cross-sectional	Japan	6000 (46.4)	≥20	In participants aged ≥60 years, the higher the household income, the higher the diet quality score (low income: 47.0, 95% CI=45.9, 48.0; middle income: 48.8, 95% CI=48.2, 49.4; high income: 49.3, 95% CI=48.1, 50.4, p=0.004 for trend in males; low income: 50.6, 95% CI=49.7, 51.6; middle income: 51.7, 95% CI=51.1, 52.3; high income: 51.2, 95% CI=50.0, 52.3, p=0.020 for trend in females).	Residential block, population size, household size, children under 15 years, occupation, BMI, smoking, and physical activity.
Yu et al., 2022 [44]	Family income	DDS	Longitudinal	China	13728 (48.3)	65-105	Family income is positively associated with DDS (β=0.2917, p<0.001 in urban area; β=0.2421, p<0.001 in rural area).	Education (literacy/illiteracy), perceived income, residence, age, cohort, sex, marital status, former occupation, multimorbidity, and self-reported health.
Segura-Badilla et al., 2021 [32]	Socioeconomic status	HEI-2010	Cross-sectional	Chile	364 (18.4)	≥60	Socioeconomic status is not associated with decreased quality of diet.	Sex, age, BMI, waist circumference, cardiovascular risk, food safety scale, obesity (BMI ≥30), food insecurity, sleep hours, and physical activity.
Chalermsri et al., 2022 [33]	Economic status	DDS	Cross-sectional	Thailand	7300 (44.1)	≥60	Economic status is associated with DDS (reference group, poorest; poor: β=0.97, 95% CI=0.70, 1.24; average: β=0.30, 95% CI=0.06, 0.54; rich: β=0.56, 95% CI=0.32, 0.80; richest: β=0.81, 95% CI=0.55, 1.06).	Age, sex, educational level, living alone, and residential area.
Yu et al., 2022 [44]	Perceived income status	DDS	Longitudinal	China	13728 (48.3)	65–105	Perceived income status is positively associated with DDS (β=0.2638, p<0.001 in urban area; β=0.4063, p<0.001 in rural area).	Education (literacy/illiteracy), family income, residence, age, cohort, sex, marital status, former occupation, multimorbidity, and self-reported health.
Nishinakagawa et al., 2023 [34]	SFS	Balanced meal consumption	Cross-sectional	Japan	8468 (43.3)	40–97	In older adults, poor SFS is positively associated with balanced meal consumption (PR=1.24, 95% CI=1.09, 1.43).	Sex, age, living arrangement, educational attainment, marital status, current work status, number of comorbidities, 6-item KPDS, and IADL.
Nishinakagawa et al., 2023 [34]	SFS × education (interaction)	Balanced meal consumption	Cross-sectional	Japan	8468 (43.3)	40–97	In older adults, there is an interactive effect of education and SFS on a low frequency of balanced meal consumption (p<0.001).	Sex, age, living arrangement, educational attainment, SFS, marital status, current work status, number of comorbidities, 6-item KPDS, and IADL.
Vaudin et al., 2021 [28]	Marital status	HEI-2010	Cross-sectional	USA	3056 (49.2)	≥60	Marital status is not associated with diet quality.	Age, race/ethnicity, education, household size, self-reported health, food expenditure, nutrition awareness/use, and food security.
Teixeira et al., 2019 [29]	Marital status	MDP	Cross-sectional	Portugal	1407 (42.5)	≥65	Marital status (married or common-law marriage) is related with increased odds of adherence to the MDP (OR=1.54, 95% CI=1.20, 1.97).	Sex, age, regional area, education, household income, and residence.
Silva et al., 2019 [30]	Marital status	Intake of dietary fibers	Cross-sectional	Brazil	1509 (40.5)	≥60	Marital status (with partner) is associated with inadequate dietary fiber consumption (PR=1.07, p=0.002).	Sex, age, income, number of chronic diseases, physical activity, smoking, desire to change weight, and sleep hours/day.
Evedove et al., 2020 [35]	Marital status	Irregular consumption of fruits and/or vegetables and legumes (i.e., ≤4 days/week)	Cross-sectional	Brazil	11185 (100)	≥60	There is no association between marital status and irregular consumption of fruits and/or vegetables.	Educational level, age group, and race/skin color.
Son et al., 2019 [36]	Living alone and eating alone (combined)	Intakes of energy and nutrients (carbohydrate, protein, lipids)	Cross-sectional	South Korea	16015 (not stated	≥19	Among older adults, none of the dietary factors differ significantly by living arrangements and eating behavior.	Age, sex, income, education, smoking, alcohol consumption, and physical activity.
Laursen et al., 2019 [42]	Household composition	Intakes of fish, red meat, and fruits/vegetables	Cross-sectional	Denmark	85456 (45.2)	≥25	Older males living with others have significantly higher intakes of fish, red meat, and fruits/vegetables than participants living alone. Older females living with others have significantly higher intakes of fish and red meat than participants living alone.	Educational level and age.
Chalermsri et al., 2022 [33]	Household composition	DDS	Cross-sectional	Thailand	7300 (44.1)	≥60	Living alone is negatively associated with DDS (β=-0.27, 95% CI=-0.53, 0.00, p=0.049).	Age, sex, educational level, wealth index, and residential area.
Vaudin et al., 2021 [28]	Educational level	HEI-2010	Cross-sectional	USA	3056 (49.2)	≥60	Education status (some college/college+) is associated with diet quality in males (β=5.3, 95% CI=3.0, 7.6) and females (β=2.1, 95% CI=0.2, 4.0).	Age, race/ethnicity, marital status, household size, self-reported health, food expenditure, nutrition awareness/use, and food security.
Teixeira et al., 2019 [29]	Educational level	MDP	Cross-sectional	Portugal	1407 (42.5)	≥65	Higher educational level (≥5 years) is associated with increased odds of adherence to the MDP (OR=2.38, 95% CI=1.54, 3.69).	Sex, age, regional area, marital status, household income, and residence.
Hashimoto et al., 2021 [37]	Educational level	Japanese Food Guide Spinning Top scores	Cross-sectional	Japan	5976 (0)	34–94	The mean diet quality score is higher in participants with high or middle education than in those with low education (low education: mean=49.8, 95% CI=49.3, 50.3; middle education: mean=50.8, 95% CI=50.3, 51.3; high education: mean=52.2, 95% CI=51.1, 53.2). In comparison with those with low education, participants with high and middle education had better scores of “sodium from seasonings” (low education: mean=5.8, 95% CI=5.7, 6.0; middle education: mean=6.1, 95% CI=6.0, 6.3; high education: mean=7.0, 95% CI=6.7, 7.4) and “fruits” (low education: mean=5.0, 95% CI=4.8, 5.2; middle education: mean=5.4, 95% CI=5.2, 5.6; high education: mean=6.0, 95% CI=5.6, 6.4).	Age, BMI, living status, marital status, smoking, physical activity, prescription medication, diet cost, urban-rural classification, primary sector employment %, areal deprivation index, number of food retailers, and region.
Chalermsri et al., 2022 [33]	Educational level	DDS	Cross-sectional	Thailand	7300 (44.1)	≥60	Educational level is positively associated with DDS (primary education: β=0.76, 95% CI=0.52, 1.03; secondary or higher education: β=1.37, 95% CI 1.04, 1.70).	Age, sex, living alone or not, wealth index, and residential area.
Nishinakagawa et al., 2023 [34]	Educational level	Balanced meal consumption	Cross-sectional	Japan	8468 (43.3)	40–97	In older adults, lower educational attainment is positively associated with a low frequency of balanced meal consumption (PR=1.14, 95% CI=1.00, 1.30).	Sex, age, living arrangement, subjective financial status, marital status, current work status, number of comorbidities, 6-item KPDS, and IADL.
Yu et al., 2022 [44]	Literacy	DDS	Longitudinal	China	13728 (48.3)	65–105	Literacy (vs. illiteracy) is positively associated with DDS (β=0.1645, p=0.0003 in urban area; β=0.0932, p=0.0080 in rural area).	Family income, perceived income, residence, age, cohort, sex, marital status, former occupation, multimorbidity, and self-reported health.
**Psychological factors**								
Elstgeest et al., 2019A [38]	Depressive symptoms (current, chronic, and history)	MDS, AHEI-2010, and DASH	Cross-sectional	Netherlands	1312 (48.1)	Median 65.1	In males, current depressive symptoms (CES-D≥16) are associated with lower MDS (β=-1.21, 95% CI=-2.41, -0.02) and AHEI (β=-2.72, 95% CI=-5.24, -0.20) scores. Chronic depressive symptoms (CES-D ≥16 in 2011–2013 and 2015/2016 versions) are associated with lower MDS scores (β=-2.22, 95% CI=-3.40, -1.04) and a trend for lower AHEI scores (β=-2.37, 95% CI=-4.92, 0.18). In males, history of depressive symptoms (ever CES-D ≥16 from 2001–2003 to 2011–2013) is associated with lower MDS (β=-1.87, 95% CI=-3.47, -0.27) and AHEI (β=-4.33, 95% CI=-7.54, -1.13) scores.	Age, cohort, educational level, marital status, physical activity, smoking, and number of chronic diseases.
Elstgeest et al., 2019B [45]	Depressive symptoms	Intake of foods (vegetables, nuts and legumes, potatoes, dairy products, fish and shellfish, sweet foods, coffee and tea, wholegrain bread, savory snacks, and sugar-sweetened beverages and fruits juices)	Longitudinal	Italy	1058 (45.3)	20–102	Higher CES-D scores are associated with decreases in intakes of vegetables (% of change in the follow-up=0.995, 95% CI=0.990, 0.999) and red and processed meat (β=-0.006, 95% CI=-0.010, -0.001), increases in dairy product intake (% of change in the follow-up=1.008, 95% CI=1.004, 1.013), and increased odds of eating savory snacks (OR: 1.012, 95% CI=1.000, 1.024).	Baseline food group intake, age, sex, marital status, educational level, physical activity, smoking, IADL disabilities, alcohol intake, and number of chronic diseases.
**Behavioral factors**								
Pourebrahim et al., 2024 [39]	Food security status	DDS	Cross-sectional	Iran	583 (47.9)	60–80	There is no association between food insecurity and DDS.	Age, sex, household income per month, educational level, employment status, living arrangement, and residential area.
Segura-Badilla et al., 2021 [32]	Food security status	HEI-2010	Cross-sectional	Chile	364 (18.4)	≥60	Food insecurity is not associated with decreased quality of diet.	Sex, age, BMI, waist circumference, cardiovascular risk, food safety scale, socioeconomic status (vulnerable/poor), obesity (BMI ≥30), sleep hours, and physical activity.
Odunitan-Wayas et al., 2021 [40]	Food security status	Intakes of energy, nutrients (protein, total fat, total carbohydrate), and foods (fruits, vegetables, cooked porridge, starchy grains, legumes, nuts and seeds, milk and dairy products, animal protein foods, sugar and sugary foods, fats and oils, savory snacks, dished and sauces, alcohol)	Cross-sectional	South Africa	122 (0)	60–85	For nutrient intake (% of total energy from macronutrients), participants with food security have higher fat intake and lower carbohydrate intake than those with food insecurity (fat intake: 23.5% energy vs. 19.0% energy, p=0.003; carbohydrate intake: 63.2% energy vs. 67.8% energy, p=0.013). For food intake (% of total energy from foods), participants with food security have lower legume intake (1.4 vs. 2.1, p=0.049) and higher fat and oil intake (4.3 vs. 2.3, p=0.023) than those with food insecurity.	None.
Vaudin et al., 2021 [28]	Food security status	HEI-2010	Cross-sectional	USA	3056 (49.2)	≥60	Household food security (full food security) is associated with diet quality in females (β=4.0, 95% CI=1.4, 6.6).	Age, race/ethnicity, education, marital status, household size, self-reported health, food expenditure, and nutrition awareness/use.
Vaudin et al., 2021 [28]	Nutrition awareness and information use	HEI-2010	Cross-sectional	USA	3056 (49.2)	≥60	Nutrition awareness and nutrition information use are associated with diet quality in males (β=6.0, 95% CI=0.7, 11.4) and females (β=4.2, 95% CI=1.6, 6.8).	Age, race/ethnicity, education, marital status, household size, self-reported health, food expenditure, and nutrition awareness/use.
Tani et al., 2020 [41]	Cooking skill	Frequency of vegetable and fruit intakes	Cross-sectional	Japan	19378 (47.2)	≥65	In females, participants with moderate to low level of cooking skills have lower frequency of vegetable and fruit intake than those with high level of cooking skills (PR=1.61, 95% CI=1.36, 1.91).	Age, education, annual normalized household income, marital status, and medical treatment (cancer, heart disease, stroke, diabetes, hypertension, and hyperlipidemia).

Abbreviations: ADL, activities of daily living; AHEI, alternative healthy eating index; BMI, body mass index; CES-D, Center for Epidemiologic Studies Depression Scale; CI, confidence interval; DASH, Dietary Approaches to Stop Hypertension diet; DDS, Dietary Diversity Score; DII, Dietary Inflammatory Index; DQI-I, Diet Quality Index-International; DVS, Dietary Variety Score; EDI, Elderly Dietary Index; FD, functional dentition; FTUs, functional tooth units; HEI, Healthy Eating Index; IADL, Instrumental Activities of Daily Living; KPDS, Kessler Psychologic Distress Scale; MDP, Mediterranean dietary pattern; MDS, Mediterranean diet score; NRV, nutrient reference value; OR, odds ratio; OSA, oral stereognostic ability; POSA, posterior occlusal support area; PR, prevalence ratio; RR, relative risk; SFS, subjective financial status; SSFR, stimulated salivary flow rate.

### 3.3 Association between physical factors and dietary intake

Eleven studies examined oral function [[18], [19], [20], [21], [22], [23], [24], [25], [26], [27],^44^], assessed using various methods, including tooth number, functional tooth units (FTUs), repulsive force, masticatory ability, and stimulated salivary flow rate [[18], [19], [20], [21], [22], [23], [24], [25], [26], [27],^44^]. Common confounders included sex, age, smoking, alcohol intake, physical activity, body mass index (BMI), comorbidities, education, and economic status. Shen et al. studied 54,796 participants (mean age, 87.9±11.5 years) and found that those with <20 natural teeth had lower dietary diversity scores (DDSs) than those with ≥20 natural teeth [22]. Iwasaki et al. reported that, in a subgroup of 2,407 participants aged 45–98 years, including those aged ≥75 years, an increase in FTUs was associated with a lower dietary inflammatory index (i.e., a diet with high anti-inflammatory potential) [18]. In 651 adults aged ≥60 years, Gaewkhiew et al. found that 12-month changes in three dietary patterns did not differ by functional dentition (FD) status [44]. Kimble et al. also reported no association between dental status and oral health problems (combined) and dietary quality as assessed using the Elderly Dietary Index or the Healthy Eating Index (HEI) in two cohorts from the UK and the USA [27].

Shen et al. reported that adults with <20 natural teeth had lower odds of regularly consuming of fresh fruits, fresh vegetables, meat, fish and aquatic products, eggs, legumes, preserved vegetables, tea, and garlic but higher odds of consuming sugar and sweet vegetables compared with those with ≥20 teeth [22]. Lin et al. conducted a cross-sectional study to examine whether the number of natural teeth or the posterior occlusal support area (POSA) was related to the number of foods that could be consumed (five types of fruits, four types of seafood and meats, and five types of vegetables). They reported that those with <20 natural teeth tended to have lower vegetable intake, and that the greater the reduction in POSA were associated with lower intakes of fruits, vegetables, seafood, and meat [24]. Fukutake et al. investigated the association among occlusal force, oral stereognostic ability (OSA), and food intake and reported that bite force was positively associated with seafood intake; the OSA score was also positively associated with green and yellow vegetable intake [25].

In a study of 84 participants aged 90 years, Karawekpanyawong et al. reported that stimulated salivary flow rate was positively correlated with intakes of vitamins A, B2, γ-tocopherol, and n-6 fatty acids and negatively correlated with carbohydrate intake [26]. They also found that the number of teeth positively correlated with the intakes of calcium, folate, beta-carotene, and vitamins C and K [26]. Kida et al. reported that having fewer teeth was associated with higher saturated fatty acids intake [21]. Gaewkhiew et al. found higher intake of dietary fiber and vitamin B1 among participants with FD than among those without FD or dentures [23]. Fukutake et al. reported that OSA scores were positively associated with α-tocopherol intake [25]. Karawekpanyawong et al. found that participants with a chewing capacity of <173 mg/dL had lower intakes of folate and vitamin A than participants with a chewing capacity of ≥173 mg/dL [26]. One study on physical activity was identified, and it showed that participation in exercise and recreational activities reduced the relative risk of undesirable eating habits [43].

### 3.4 Association between social factors and dietary intake

Eight articles on economic status were included [[28], [29], [30], [31], [32], [33], [34],^45^]. Household income was the most commonly used measure of economic status, followed by household food expenditure, socioeconomic status, and subjective financial status [[28], [29], [30], [31], [32], [33], [34],^45^]. The main confounding factors addressed in these studies were sex, age, race, self-reported health, and residence area. The assessment indices for dietary quality varied across studies. Studies that reported a negative association between low economic status and dietary quality included those by Vaurin et al., who used the HEI-2020 [28]; Kurotani et al., who used the Japanese Food Guide Spinning Top scores [31]; Yu et al. and Chalermsri et al., who used the DDS [33,45]; and Nishinakagawa et al., who defined a balanced meal as eating a meal consisting of a staple food, main dish, and side dish at least twice daily, almost every day [34]. Meanwhile, two studies reported no link between low economic status and dietary quality: Teixeira et al. found no association between household income and adherence to the Mediterranean dietary pattern in 1,407 Portuguese adults aged ≥65 years [29], and Segura-Badilla et al. found no association between socioeconomic status and poor dietary quality (HEI-2010) in 354 Chilean adults aged ≥60 years [32].

One study examined the association between economic status and dietary fiber and reported that higher household income was associated with inadequate dietary fiber intake [30]. There were seven studies on marital status and household size [[28], [29], [30],^33^,^35^,^36^,^42^]. Three of these studies examined dietary quality [28,30,33]: one found no association with marital status [28], whereas another reported that being married or in a common-law marriage was associated with higher adherence to MDP [30], and living alone was negatively correlated with DDSs [33]. Regarding food groups, a study investigating the frequency of vegetable and fruit intake found no correlation between marital status and the frequency of vegetable and fruit intake [35]. Individuals living with others were reported to have higher intakes of meat, fish, vegetables, and fruit than those living alone [42]. In the context of nutritional intake, one study reported that living alone or eating alone is not associated with nutrient intake [36].

Six studies on education were included [28,29,33,34,37,45]. All six studies concluded that low education levels were associated with poor dietary quality [28,29,33,34,37,45]. One study examined the association between education and food groups and found that higher levels of education were associated with higher fruit intake [37].

### 3.5 Association between psychological factors and dietary intake

Although many studies examined dietary intake as an explanatory variable for depression, only two studies met the objectives of this review, including depression as an explanatory variable and dietary intake as an objective variable [38,46]. The main confounding factors addressed in these studies were sex, age, education, marital status, physical activity, smoking, and comorbidities. Elstgeest et al. evaluated dietary quality using three indices: Mediterranean diet, alternative healthy eating index (AHEI)-2010, and the Dietary Approaches to Stop Hypertension diet. They showed that depressive symptoms, as assessed using the Center for Epidemiologic Studies Depression Scale (CES-D), were associated with lower MDS scores and AHEI scores [38]. In another longitudinal study by Elstgeest et al., an increased CES-D score was associated with an increased odds ratio for decreased intakes of vegetables and red/processed meat and increased intake of dairy products and salty snacks [46]. No studies examined cognitive function with dietary intake as the outcome; therefore, none were included.

### 3.6 Association between behavioral factors and dietary intake

Four studies examined food security [28,32,39,40], adjusting for sex, age, economic status, education, and marital status. Of the three studies examining the association between food security and dietary quality, two studies found no significant association [32,39], whereas one study reported high dietary quality among females with food security [28]. Regarding food groups, one study reported that food insecurity was positively associated with legume and carbohydrate intake and negatively associated with oil, fat, and lipid intake [40]. One study focused on nutritional knowledge reported that high nutritional awareness and information use were positively associated with dietary quality as assessed by the HEI-2010 [28]. One study on cooking skills reported that among females, those with moderate to low levels of cooking skills consumed vegetables and fruits less frequently than did those with high levels of cooking skills [41].

### 3.7 Risk of bias

Table 2 shows the quality of the cohort studies as assessed using the NOS. In all longitudinal studies included, dietary intake was based on self-report tools, such as the Food Frequency Questionnaire; thus, none of the studies could be given a star in the “Outcome” No. 6 rating. The study by Abe et al. [43] was not awarded a star in the “Selection” No. 3 rating because exposure variables were assessed based on a self-administered questionnaire. Quality assessment showed that all four of the cohort studies included in the review were of a high standard [[43], [44], [45], [46]]. Table 3 shows the quality of the cross-sectional studies as assessed using the AXIS. Eighteen studies were deemed high quality [18,19,22,24,[26], [27], [28], [29], [30], [31], [32], [33], [34], [35], [36],^39^,^41^,^42^], five were moderate quality [20,21,23,25,37] and two were low quality [38,40]. Few studies reported sample size calculations, and some had limited population representativeness. Approximately half of the studies raised concerns about bias, including failure to describe characteristics of excluded participants or high exclusion rates.

**Table 2:** Quality appraisal of the included cohort studies - Newcastle-Ottawa Scale.

	Selection				Comparability	Outcome				
Authors, year of publication	1	2	3	4	5	6	7	8	Total score	Study quality
Abe et al., 2022 [43]	*	*		*	**		*	*	7	High
Gaewkhiew et al., 2021 [44]	*	*	*	*	**		*	*	8	High
Yu et al., 2022 [45]	*	*	*	*	**		*	*	8	High
Elstgeest et al., 2019 [46]	*	*	*	*	**		*	*	8	High

1) Representativeness of the exposed cohort

2) Selection of the non-exposed cohort

3) Ascertainment of exposure

4) Demonstration that the outcome of interest was not present at the start of the study

5) Comparability of cohorts on the basis of the design or analysis controls for confounders

6) Assessment of outcome

7) Was the follow-up long enough for outcomes to occur

8) Adequacy of follow-up of cohorts

**Table 3:** Quality appraisal of the included cross-sectional studies - appraisal tool for cross-sectional studies.

	Introduction	Methods										Results					Discussion		Other			
Authors, year of publication	1	2	3	4	5	6	7	8	9	10	11	12	13	14	15	16	17	18	19	20	Total score	Study quality
Iwasaki et al., 2024 [18]	Y	Y	N	Y	Y	Y	P	Y	Y	Y	Y	Y	N	N	Y	Y	Y	Y	Y	Y	16	H
Milledge et al., 2021 [19]	Y	Y	N	Y	Y	N	P	Y	Y	Y	Y	Y	Y	P	Y	Y	Y	Y	Y	Y	16	H
Kusama et al., 2023 [20]	Y	Y	N	Y	P	N	N	Y	Y	Y	Y	Y	Y	N	Y	Y	Y	Y	Y	Y	15	M
Kida et al., 2023 [21]	Y	Y	N	Y	N	N	N	Y	Y	Y	Y	Y	Y	N	Y	Y	Y	Y	Y	Y	15	M
Shen et al., 2023 [22]	Y	Y	Y	Y	Y	Y	N	Y	Y	Y	Y	Y	N	N	Y	Y	Y	Y	Y	Y	17	H
Gaewkhiew et al., 2019 [23]	Y	Y	Y	Y	P	?	N	Y	Y	Y	Y	Y	N	P	Y	Y	Y	Y	Y	Y	15	M
Lin et al., 2021 [24]	Y	Y	Y	Y	P	Y	Y	Y	Y	Y	Y	Y	N	N	Y	Y	Y	Y	Y	Y	17	H
Fukutake et al., 2019 [25]	Y	Y	Y	Y	P	N	N	Y	Y	Y	Y	Y	N	N	Y	Y	Y	Y	Y	Y	15	M
Karawekpanyawong et al., 2023 [26]	Y	Y	N	Y	N	N	Y	Y	Y	Y	Y	Y	Y	P	Y	Y	Y	Y	Y	Y	16	H
Kimble et al., 2023 [27]	Y	Y	N	Y	P	Y	N	Y	Y	Y	Y	Y	Y	N	Y	Y	Y	Y	Y	Y	16	H
Vaudin et al., 2021 [28]	Y	Y	N	Y	Y	Y	P	Y	Y	Y	Y	Y	Y	N	Y	Y	Y	Y	Y	Y	17	H
Teixeira et al., 2019 [29]	Y	Y	Y	Y	Y	N	Y	Y	Y	Y	Y	Y	N	Y	Y	Y	Y	Y	Y	Y	18	H
Silva et al., 2019 [30]	Y	Y	N	Y	Y	Y	Y	Y	Y	Y	Y	Y	N	N	Y	Y	Y	Y	Y	Y	17	H
Kurotani et al., 2020 [31]	Y	Y	N	Y	Y	Y	P	Y	Y	Y	Y	Y	Y	N	Y	Y	Y	Y	Y	Y	17	H
Segura-Badilla et al., 2021 [32]	Y	Y	Y	Y	Y	N	N	Y	Y	Y	Y	Y	?	N	Y	Y	Y	Y	Y	Y	16	H
Chalermsri et al., 2022 [33]	Y	Y	N	Y	Y	Y	Y	Y	Y	Y	Y	Y	Y	Y	Y	Y	Y	Y	Y	Y	19	H
Nishinakagawa et al., 2023 [34]	Y	Y	N	Y	Y	Y	N	Y	Y	Y	Y	Y	N	Y	Y	Y	Y	Y	Y	Y	17	H
Evedove et al., 2020 [35]	Y	Y	N	Y	Y	Y	P	Y	Y	Y	Y	Y	Y	N	Y	Y	Y	Y	Y	Y	17	H
Son et al., 2019 [36]	Y	Y	N	Y	Y	Y	P	Y	Y	Y	Y	Y	Y	N	Y	Y	Y	Y	Y	Y	17	H
Hashimoto et al., 2021 [37]	Y	Y	N	Y	N	N	N	Y	Y	Y	Y	Y	Y	N	Y	Y	Y	Y	Y	Y	15	M
Elstgeest et al., 2019 [38]	Y	Y	N	Y	P	P	N	Y	Y	Y	Y	Y	P	N	Y	Y	Y	Y	Y	Y	14	L
Pourebrahim et al., 2024 [39]	Y	Y	Y	Y	Y	Y	Y	Y	Y	Y	Y	Y	N	N	Y	Y	Y	Y	Y	Y	18	H
Odunitan-Wayas et al., 2021 [40]	Y	Y	N	Y	N	N	N	Y	Y	Y	Y	Y	?	N	Y	Y	Y	Y	Y	Y	14	L
Tani et al., 2020 [41]	Y	Y	N	Y	Y	Y	N	Y	Y	Y	Y	Y	Y	N	Y	Y	Y	Y	Y	Y	17	H
Laursen et al., 2019 [42]	Y	Y	Y	Y	Y	P	P	Y	Y	Y	Y	Y	P	N	Y	Y	Y	Y	Y	Y	16	H

Key symbols: Y=Yes, N=No, P=Partially, ?=No information available to make a decision, H=high, M=moderate, L=low.

1) Were the aims/objectives of the study clear?

2) Was the study design appropriate for the stated aim(s)?

3) Was the sample size justified?

4) Was the target/reference population clearly defined? (Is it clear who the research was about?)

5) Was the sample frame taken from an appropriate population base so that it closely represented the target/reference population under investigation?

6) Was the selection process likely to select subjects/participants that were representative of the target/reference population under investigation?

7) Were measures undertaken to address and categorize non-responders?

8) Were the risk factor and outcome variables measured appropriately to the aims of the study?

9) Were the risk factor and outcome variables measured correctly using instruments/measurements that had been trialed, piloted, or published previously?

10) Is it clear what was used to determine statistical significance and/or precision estimates (e.g., p-values, confidence intervals)?

11) Were the methods (including statistical methods) sufficiently described to enable them to be repeated?

12) Were the basic data adequately described?

13) Does the response rate raise concerns about non-response bias?

14) If appropriate, was information about non-responders described?

15) Were the results internally consistent?

16) Were the results presented for all the analyses described in the methods?

17) Were the authors’ discussions and conclusions justified by the results?

18) Were the limitations of the study discussed?

19) Were there any funding sources or conflicts of interest that may affect the authors’ interpretation of the results?

20) Was ethical approval or consent of participants attained?

## 4 Discussion

This is the first study to systematically map food and nutrient intake patterns across four domains (physical, social, psychological, and behavioral) among community-dwelling older adults and to identify vulnerabilities in specific nutrients and food groups within each domain. Although the included literature was limited, the review found that poor oral function, depressive symptoms, and low education and economic status are associated with low intake of protein- and micronutrient-rich foods and with poor dietary quality. These dietary changes may accelerate sarcopenia, frailty, and functional decline, creating a “vicious cycle” of reduced appetite, capacity, and intake. Anorexia of aging manifests this cycle and further accelerates it [3]. Therefore, these findings can inform the development of interventions to maintain older adults’ health. However, evidence describing food and nutrient intakes among individuals with cognitive decline, poor cooking skills or nutritional knowledge, or diminished taste and smell was limited. Future research should address gaps, such as identifying which foods and nutrients are consumed less by individuals with poor cooking skills or limited nutritional knowledge.

### 4.1 Influence of physical, social, psychological, and behavioral factors on dietary intake

Poor oral function was associated with lower intake of protein-rich foods (e.g., meat, fish, and beans) as well as vegetables and fruits rich in vitamins, minerals, antioxidants, and dietary fiber [[21], [22], [23], [24], [25], [26]]. Previous studies show that dietary guidance for individuals with poor oral function can improve nutritional intake [47,48]. However, associations between oral function and dietary quality were inconsistent [18,22,27,44], possibly attributable to heterogeneity in assessment methods for oral function and dietary quality. The findings of this review may inform guidance strategies, such as cooking techniques that make these foods easier to chew for individuals with reduced oral function.

Economic status was not examined in relation to specific food or nutrient intake in the included studies, although most assessed its association with dietary quality. Among these, 75% reported that low economic status was linked to poorer dietary quality [28,31,33,34,45], whereas two studies found no association [29,32]. Teixeira et al. suggested that the null finding may reflect substantial income non-reporting [29]. In contrast, Segura-Badilla et al. reported that although 82.4% of participants had poor economic circumstances, 65.7% maintained food security, indicating that food insecurity strongly influenced dietary quality [32]. This suggests that ensuring physical and economic access to food may help prevent declines in dietary quality even among economically disadvantaged older adults.

Marital or living-alone status showed no consistent association with dietary quality or food intake [28,30,33,35,36,42], possibly attributable to differences in sex, cooking skills, nutritional knowledge, and attitudes toward nutrition [49]. As only one study examined cooking skills and dietary intake, further reviews focusing on household status and cooking skills are warranted. Identifying poor cooking skills and food groups with likely intake declines may help inform support measures, such as simplified cooking methods for specific foods. Although culinary nutrition education programs can improve eating habits and health literacy in older adults, few have been specifically designed for this population [50]. Increased research on cooking skills and dietary intake is therefore needed to inform optimal program development.

Low educational level was consistently associated with poor dietary quality [28,29,33,34,37,45], likely through its influence on food choices and access to health information [49]. Nutritional education interventions have been shown to increase vegetable, fruit, and dietary fiber intakes in older adults [51], suggesting particular importance for those with lower educational backgrounds. However, only one study examined associations with specific food groups [37], highlighting the need for further research to identify foods most affected by low educational level.

Few studies examined depression and dietary intake, but those that did found insufficient intake among individuals with depression [38], characterized by lower meat and vegetable intake and higher dairy and salty snacks consumption [46]. Depression may reduce appetite in older adults [52,53]. Older individuals tend to prioritize preferred foods and sensory appeal [54]. Thus, interventions that stimulate food cravings, alongside depression treatment, may be necessary. None of the included studies examined cognitive function and dietary intake; however, cognitive decline may restrict behaviors such as cooking and shopping, particularly in the absence of a supportive partner. Further studies are needed to clarify these associations.

Behavioral constraints include low nutritional knowledge, reduced shopping ability, poor food access, and limited cooking skills, yet few studies have characterized dietary intake based on these factors. In addition, no standardized indicators exist to assess nutritional knowledge or cooking skills. Given their influence on dietary intake in older adults [54], further research in this area is needed.

### 4.2 Clinical and practical implications

Our findings highlight the importance of primary care and community health clinicians assessing older adults while considering links between diet and physical, social, and psychological limiting factors. Tools that can easily assess these factors include Intrinsic Capacity [55], the Kihon Checklist [56], and the Frailty-Intrinsic Capacity index [57]. Dietary diversity can be readily assessed using the DDS [58].

The results also suggest that older individuals with poor oral function, low economic or educational status, and depressive symptoms should be evaluated and supported by dietitians. Dietitians need to understand the poor eating habits associated with these limiting factors and provide tailored support to optimize dietary intake. For example, meat and vegetables can be prepared in forms that are easier to chew, such as by grinding or boiling. Dietitians can also offer simple, low-cost recipes and shopping support for individuals with limited socioeconomic resources or cooking skills. In collaboration with psychologists, integrated nutrition–mental health interventions may be provided for those with depressive symptoms.

### 4.3 Limitations and future research

This study has some limitations. First, the literature search was limited to the past 5 years to capture recent evidence, which may have reduced the number of eligible studies. Second, only studies published in Japanese or English were included, potentially excluding relevant research in other languages. Third, most included studies were cross-sectional, with few longitudinal studies examining oral function (n=1), economic factors (n=1), or depression (n=1); therefore, causal relationships cannot be inferred. For example, poor diet may be a risk factor for depression and poor oral function [59,60]. However, because this review aimed to characterize dietary patterns among older adults already experiencing limiting factors, cross-sectional evidence was considered sufficient. Nevertheless, future longitudinal studies are needed to identify strategies to prevent poor dietary habits. Fourth, this study was designed as a comprehensive qualitative review to systematically examine how physical, social, psychological, and behavioral exposure factors influence food and nutrient intake. Comparisons across studies were challenging because of heterogeneity in dietary assessment methods, dietary pattern definitions, and measures of dietary quality. Despite these challenges, the review qualitatively summarized nutrients and food groups at risk of imbalance for each exposure factor, providing valuable guidance for future nutritional support strategies for older adults.

This study also has important implications for future research. First, further studies are needed to better characterize behavioral and psychological constraints and their associations with dietary habits, as well as to develop standardized measures for assessing cooking skills and nutritional knowledge. Second, our findings support the design of intervention studies targeting older adults with poor oral function, lower educational or economic status, or depressive symptoms. Third, as evidence accumulates, this work will also inform future research on risk identification and interventions that integrate multidimensional assessments (frailty, sarcopenia, cognitive decline, and anorexia) with detailed dietary evaluations.

## 5 Conclusion

Reduced oral function and depressive symptoms are associated with lower intake of protein-rich foods (e.g., meat, fish, and legumes) and of vegetables and fruits rich in vitamins, minerals, and dietary fiber. Low economic status and educational level are associated with poor dietary quality. However, evidence linking many factors to specific nutrients or food groups remains limited. In particular, few studies have examined the effects of cooking techniques, nutritional knowledge, or diminished taste and smell on dietary intake. Although these findings may inform the development of support measures and educational programs for older adults, they also highlight substantial gaps in research on dietary intake characteristics associated with multiple limiting factors.

## Ethical statement

This study is a systematic review of previously published research and did not involve the collection of new data from human participants or animals. Therefore, ethical approval, informed consent, and study registration were not required. This article does not contain any studies involving human participants or animals performed by the authors.

## Funding

This work was supported by the Health Labor Sciences Research [grant number 24FA1012]. The funding sources had no involvement in the study design, collection, analysis and interpretation of data, writing of the report, or decision to submit the article for publication.

## Declaration of Generative AI and AI-assisted technologies in the manuscript preparation process

During the preparation of this work, the authors used DeepL in order to proofread the paper, but did not use AI for any other purposes (e.g., to search for papers to review). After using DeepL, the authors reviewed and edited the content as needed and take full responsibility for the content of the published article.

## Data statement

This study is based on previously published literature. All data supporting the findings are available from the cited sources.

## CRediT authorship contribution statement

**Kaori Kinoshita:** Writing – review & editing, Writing – original draft, Methodology, Investigation, Funding acquisition, Data curation, Conceptualization. **Kanae Furuya:** Writing – review & editing, Methodology, Investigation, Data curation. **Ryoko Katagiri:** Writing – review & editing, Project administration, Methodology, Investigation, Funding acquisition.

## Declaration of competing interest

The authors declare the following financial interests/personal relationships which may be considered as potential competing interests:

Ryoko Katagiri reports financial support was provided by Health Labor Sciences Research. If there are other authors, they declare that they have no known competing financial interests or personal relationships that could have appeared to influence the work reported in this paper.

## Appendix Supplementary materials

Image, application 1
