# Supplementary material for: Dietary intake characteristics in older adults: A systematic review of physical, social, psychological, and behavioral limitations
Source: JAR Life. 2026 Feb 6;15:100064. doi: 10.1016/j.jarlif.2026.100064 (PMC12907051; doi:10.1016/j.jarlif.2026.100064)
Supplement: Supplementary file 1 [file mmc1.pdf]

**MEDLINE**

(“aged”[MeSH Terms] OR "older adults" OR "older people" OR "older person" OR "elderly" OR "senior") AND ((“oral health”[MeSH Terms] OR "oral problem" OR "oral condition" OR "oral frail" OR "oral dysfunction" OR "tooth loss"[MeSH Terms] OR “swallowing disorder” OR "chewing problem") OR (“loss of taste” OR “taste loss” OR “taste disorder”) OR (“loss of smell” OR “smell loss” OR “hyposmia”) OR (“economic status”[MeSH Terms] OR “socioeconomic factors”[MeSH Terms]) OR (“access to healthy foods”[MeSH Terms] OR “food insecurity”[MeSH Terms]) OR (“living alone” OR “home environment”[MeSH Terms] OR “eating alone”) OR (”social participation”[MeSH Terms] OR “social support”[MeSH Terms] OR “social isolation”[MeSH Terms] OR “meals on wheels” OR “home helper”) OR (“depression”[MeSH Terms] OR “cognitive function”) OR (“cooking skill” OR “nutrition knowledge” OR “food selection” OR “food preferences”[MeSH Terms])) AND (”eating”[MeSH Terms] OR “diet”[MeSH Terms] OR "food" OR "nutrients" OR "diet" OR "meal" OR "dietary intake" OR "diet quality") AND (“observational stud*”[TIAB] OR “cohort stud*”[TIAB] OR “follow-up stud*”[TIAB] OR “epidemiologic stud*”[TIAB] OR “prospective stud*”[TIAB] OR “retrospective stud*”[TIAB] OR “longitudinal stud*”[TIAB] OR “cross-sectional stud*”[TIAB]) AND ("English"[Language] OR "Japanese"[Language]) AND (2019/06/01:2024/06/01[Date - Publication])

**Web of Science**

(TS=("aged" OR "older adults" OR "older people" OR "older person" OR "elderly" OR "senior")) AND
((TS=("oral health" OR "oral problem" OR "oral condition" OR "oral frail" OR "oral dysfunction" OR "tooth loss" OR "swallowing disorder" OR "chewing problem")) OR
(TS=("loss of taste" OR "taste loss" OR "taste disorder")) OR
(TS=("loss of smell" OR "smell loss" OR "hyposmia")) OR
(TS=("economic status" OR "socioeconomic factors")) OR
(TS=("access to healthy foods" OR "food insecurity")) OR
(TS=("living alone" OR "home environment" OR "eating alone")) OR
(TS=("social participation" OR "social support" OR "social isolation" OR "meals on wheels" OR "home helper")) OR
(TS=("depression" OR "cognitive function")) OR
(TS=("cooking skill" OR "nutrition knowledge" OR "food selection" OR "food preferences"))) AND
(TS=("eating" OR "diet" OR "food" OR "nutrients" OR "meal" OR "dietary intake" OR "diet quality")) AND
(TS=("observational stud*" OR "cohort stud*" OR "follow-up stud*" OR "epidemiologic stud*" OR "prospective stud*" OR "retrospective stud*" OR "longitudinal stud*" OR "cross-sectional stud*")) AND
(LA=("English" OR "Japanese")) AND
PY=(2019-2024)
